# Radioiodine adjuvant therapy in differentiated thyroid cancer: An update and reconsideration

**DOI:** 10.3389/fendo.2022.994288

**Published:** 2022-11-30

**Authors:** Yu-qing Sun, Di Sun, Xin Zhang, Ying-qiang Zhang, Yan-song Lin

**Affiliations:** Department of Nuclear Medicine, State Key Laboratory of Complex Severe and Rare Diseases, Peking Union Medical College (PUMC) Hospital, Chinese Academy of Medical Sciences & PUMC, Beijing, China

**Keywords:** differentiated thyroid cancer, radioiodine therapy (RAI), 131I adjuvant therapy, nuclear theranostics, 131I whole-body scan

## Abstract

Radioiodine (^131^I) therapy (RAI) has been utilized for treating differentiated thyroid cancer (DTC) for decades, and its uses can be characterized as remnant ablation, adjuvant therapy (RAT) or treatment for known diseases. Compared with the definite ^131^I treatment targets for remnant ablation and known disease, ^131^I adjuvant therapy (RAT) aims to reduce the risk of recurrence by destroying potential subclinical disease. Since it is merely given as a risk with no imaging confirmation of persistence/recurrence/metastases, the evidence is uncertain. With limited knowledge and substance, the indication for RAT remains poorly defined for everyday clinical practice, and the benefits of RAT remain controversial. This ambiguity results in a puzzle for clinicians seeking clarity on whether patients should receive RAT, and whether patients are at risk of recurrence/death from undertreatment or adverse events from overtreatment. Herein, we clarified the RAT indications in terms of clinicopathological features, postoperative disease status and response to therapy evaluation, and retrospectively examined the clinical outcomes of RAT as reported in current studies and guidelines. Furthermore, given the evolution of nuclear medicine imaging techniques, it can be expected that the future of RAT may be advanced by nuclear medicine theranostics (i.e., ^131^I whole-body scan, PET/CT) by accurately revealing the biological behaviors, as well as the underlying molecular background.

## 1 Introduction

As the most common endocrine cancer, thyroid cancer’s incidence keeps rising worldwide (1). Differentiated thyroid cancer (DTC), mainly consisting of papillary thyroid cancer (PTC) and follicular thyroid cancer (FTC), accounts for 94% (2) of thyroid cancer and it generally carries a favorable clinical outcome under surgery followed by radioiodine (RAI) therapy and thyroid stimulating hormone (TSH) suppressive therapy.

Currently, management of RAI can be characterized as RAI for remnant ablation, adjuvant therapy (RAT) and treatment for known disease (3). As for clinical practice, RAI for remnant ablation is aimed at destroying residual thyroid tissue, thereby increasing the sensitivity of long-term monitoring by using serum thyroglobulin(Tg) and diagnostic radioiodine-131 whole-body scan (Dx-WBS). RAI for known disease is aimed at destroying persistently locoregional or distant metastases, in order to reduce recurrence and mortality or for palliation. Unlike the other two goals of RAI treatment that target remnant thyroid tissue or known disease, RAT is given to treat subclinical tumors that may or may not actually be present after prior adequate treatment (4).

As we noticed, the concept of RAT has been evolved over decades. In 1957, RAT was initially embodied in “ablation”, which intended to destroy both normal residual thyroid tissue and concealed microscopic tumor foci (5, 6). In 1996, the role of routine “ablation” was further specified by the 1996 American Thyroid Association Management Guidelines (the 1996 ATA Guidelines) as decreasing the risk of recurrent locoregional disease and facilitating long-term surveillance (7). It was not until 2009 that “adjuvant therapy” (RAT) was separated from remnant ablation with an aim to destroy subclinical lesions and this definition has been used ever since (3, 8). Since the existence of subclinical lesions cannot be accurately confirmed by current imaging modalities, the indication of RAT remains controversial, elusive and ambiguous, making it a dilemma during RAI decision-making.

Thus, this review intends to offer insights into clarifying RAT by integrating recently published evidence, and the indications and benefits of RAT were summarized with interpretations of three aspects: clinicopathological features, postoperative disease status, and response to therapy evaluation as follows.

### 1.1 Clinicopathological features guided RAT

#### 1.1.1 Pros and cons of clinicopathological features guided RAT

Some tumor features have a profound impact on prognosis. Current studies have indicated that patients with highly suspicious clinicopathologic findings including aggressive tumor histology, large primary tumor size, local invasion, vascular invasion and gene mutations, carry a higher risk of recurrence and are potential targets/candidates for RAT (9–11). However, the clinical benefit of RAT in these patients remains debatable (**Table 1**).

**Table 1:** Clinical outcomes of different clinicopathological features guided RAT.

Study	Study design	Number	Clinical-pathological features	Follow-up period	Outcome	Prognosis improved	Dosage	Benefited groups	P
Ruel(2015) (12)	Retrospective	21870	Intermediate risk patients, as defined by ATA risk and AJCC disease stage T3, N0, M0 or Mx and T1–3, N1, M0 or Mx.	6y	OS	Yes	Na	Intermediate risk patients, as defined by ATA risk and AJCC disease stage T3, N0, M0 or Mx and T1–3, N1, M0 or Mx.	<0.001
Al-Qahtani(2015) (13)	Retrospective	326	PTMC with aggressive histopathologic variants, multifocality, extrathyro idal extension, lymphovascular space invasion, tumor size (>0.5 cm) and lymph node involvement	median 8.05y(1.62-11.4y)	DFS	Yes	30-200mCi; 100mCi is recommended for N0	PTMC with aggressive histopathologic variants, multifocality, lymphovascular space invasion, lymphovascular space invasion, tumor size (>0.5 cm) and lymph node involvement	<0.05
Creach(2012) (14)	Retrospective	407	Size of PTMC, histological subtype, positive lymph nodes on first WBS, PTMC (≤0.8 cm), lymph node metastases, pathology, vascular invasion, capsular invasion, soft tissue invasion, or positive surgical margins.	Median 5.3y(0.2-51 y)	5-y RFS	Yes	Median 100mCi	PTMC (≤0.8 cm), lymph node metastases.	<0.05
Kim(2013) (15)	Retrospective	480	Patients with PTMC with microscopic extrathyroidal extension, cervical lymph node metastases or multifocality (Intermediate-risk)	median 5.3y(0.08-15.4y)	DFS	No	Cumulative 30-300mCi	Patients with PTMC with microscopic extrathyroidal extension, cervical lymph node metastases or multifocality (Intermediate-risk)	0.79
Ballal(2016) (16)	Retrospective	254	Patients with T1-3 tumor, N1a/1b and with no distant metastasis (M0).	10.3y	DFS	No	30-100mCi	Patients with T1-3 tumor, N1a/1b and with no distant metastasis (M0).	0.005

Here, current studies with long-term follow-up and large sample sizes are listed.

Multivariate adjusted analyses from Surveillance, Epidemiology, and End Results Program (SEER) indicated that RAT in DTC patients with aggressive histologies such as diffuse sclerosing, tall cell, and insular variants were associated with improved overall survival (OS) (9, 10). A meta-analysis found that RAT can improve the cause-specific survival in high-risk DTC patients that were aged >45 years with primary tumors >4 cm, microscopic extrathyroidal invasion, and/or lymph node metastases (6^th^ UICC/AJCC TNM stage III/IV), yet patients aged <45 years with microscopic central compartment lymph node metastases are however, failed to benefit from RAT (17). The clinical outcome was also not clear in lateral or macroscopic lymph node metastatic patients (17). Recently, a retrospective study focused on the optimal timing, demonstrated that a prolonged RAT interval (time between thyroidectomy and 1^st^ RAT ≥3 months) may cause an incomplete response (categorized by the 2015 ATA Guidelines), as well as soft tissue invasion (18). Thus, in terms of patients with aggressive histology, large primary tumor size and local/vascular invasion, definite benefits can be seen from RAT. Moreover, Ruel et al. studied 21,870 adult PTC patients who underwent total thyroidectomy with/without RAT in the National Cancer Database (NCDB) from 1998–2006, involving microscopic soft tissue invasion, positive lymph nodes, aggressive histology, vascular invasion, RAI uptake outside the thyroid gland on WBS, or T3N0M0/Mx, T3N0M0/Mx and T1–3 N1M0/Mx by 6^th^ UICC/AJCC TNM. During the median follow-up of 6 years, RAT was indicated to improve the OS, with a 29% reduction in the risk of death with a hazard ratio (HR) of 0.71, 95% confidence interval (95% CI) 0.62–0.86, p<0.001. For age < 45 years, the reduction in death risk associated with RAT was up to 36%, HR 0.64, p=0.016 (12). Moreover, fewer patients developed distant metastases (P < 0.002) after RAT, yet this effect is observed only in patients whose tumor size was more than 1.5cm (19). Even for papillary microcarcinoma (PTMC) patients, a retrospective study involving 326 patients also indicated RAT was associated with improved disease-free survival (DFS) or in those with aggressive histopathologic variants, multifocality, extrathyroidal extension, lymphovascular space invasion, tumor size (>0.5 cm) and lymph node involvement (13). Another study focused on PTMC patients with lymph node metastasis also demonstrated longer 5-year recurrence-free survival (5y-RFS) in those who underwent RAT than that in those who didn’t (93.2%vs. 42.9%) (14). In contrast, Kim et al. studied PTMC patients of complete tumor resection, no extrathyroidal extension, no cervical lymph node metastasis, no distant metastasis, microscopic extrathyroidal extension, no cervical lymph node metastases or multifocality, and suggested that those patients were unlikely to benefit from RAT (15).

Over the recent years, *BRAF^V600E^* mutation has been valued as an aggressive clinicopathological feature with a high recurrence rate and tumor-specific mortality (11), Jiao Li et al. found a noninferior response to RAT in nonmetastatic *BRAF^V600E^*-mutated PTC patients, which may suggest a possible effect of RAT in improving the general clinical outcome in this patient group (20).

However, some studies suggested that RAT guided only by clinicopathological features may lead to overtreatment in patients who have been treated sufficiently by their primary surgery. Ballal et al. studied 254 postoperative DTC patients with age >45 years, vascular invasion, microscopic extrathyroidal extension, the presence of cervical lymph node metastases and the presence of aggressive histological variants who were stratified as intermediate-risk. By integrating nuclear medicine modalities (e.g. Dx-WBS) and biochemical markers(e.g. Tg), patients showing RAI uptake ≤0.2% in the first Dx-WBS along with postoperative stimulated Tg levels (ps-Tg) ≤10 ng/ml can be spared from RAT, with similar disease-free survival rate compared with RAT group(92% vs 90%), suggesting that not all intermediate-risk patients need RAT (16).

#### 1.1.2 Recommendations from guidelines concerning the clinicopathological features guided RAT

Based on the evidence mentioned above, the role of clinicopathological features in directing RAT consideration has been highlighted by multiple guidelines (**Table 2**), including the ATA Guidelines, 2019 European Society for Medical Oncology Clinical Practice Guidelines for Thyroid Cancer (2019ESMO Guidelines), 2022 European Thyroid Association consensus statement(2022ETA Consensus Statement) and the National Comprehensive Cancer Network in Oncology Guidelines (NCCN Guidelines) (3, 21–23). From which we can see the evolution of RAT in different guidelines. For instance, the ATA Guidelines have gradually specified the clinicopathological features, and adjusted the dosage recommendations accordingly, from 100-200 mCi to 30-150 mCi, which may due to the uncertain benefit from large dosage of RAT (3). Molecular pathological features, especially *BRAF^V600E^*, were selectively suggested for RAT in the 2015 ATA Guidelines, whereas the 2019 ESMO Guidelines underscored the significance of the coexistence of the *BRAF^V600E^* and *TERT* mutations (3, 23). The 2022 ETA Consensus Statement suggested patients with advanced age, aggressive histologies, increasing volume of nodal disease, extranodal extension, multiple N1 and/or lymph node metastases outside the central neck should receive RAT. Of note, the NCCN Guidelines provided more specified indications of RAT and appeared more practical in clinical use, including suspicious clinicopathologic findings, postoperative unstimulated Tg levels and radiological examination and/or Dx-WBS (3, 21, 23, 24). From these recommendations of guidelines, an area of controversy can be seen, making it too sticky for clinicians to follow.

**Table 2:** Evolution of RAT in guidelines by clinicopathological features.

	ATA guidelines		2019 ESMO guidelines	NCCN guidelines			2022 ETA Consensus Statement
Indications	2009 ATA guidelines	2015 ATA guidelines		2018 NCCN guidelines	2020 NCCN guidelines	2021 NCCN guidelines	
*Clinicopathological features guided*	Tumor >1.5 cm	(Updated on 2009 ATA Guidelines)	Intermediate-and high-risk	Clinicopathologic findings (+), Dx-WBS (-):	(Updated on 2018NCCN Guidelines)	(Updated on 2020NCCN Guidelines)	1. High-risk^b^ *
	With post-operatively residual disease	1.1 Intermediate-risk^a^	(Updated on 2015 ATA Guidelines)	1. Tumor >2cm;	1. RAT selectively recommended:		2. Intermediate-risk^c^
		1.2 High-risk^b^ *	1. Intermediate-risk: Tumor-related symptoms	2. MVI (+)	Tumor 2–4 cm		
		2. *BRAF^V600E^*	2. High-risk: *TERT*(+) and *BRAF^V600E^* (+)	3. cLNM (+)	2. RAT recommended*:		
				4. Microscopic margins (+)	Tumor >4 cm		
				5. ETE (+)	ETE (+)		
					EVI^d^		
					Bulky or LNM >5	Dx-WBS (-), CT/MRI+	
**Dosage**	100-200 mCi	30-150 mCi		50-100 mCi	50-200 mCi	50-100mCi	
		T3&N1: the effectiveness of RAT >150 mCi is uncertain	Intermediate risk: 30-100 mCi		RAI for known disease (100-200mCi) is embodied in RAT (50-100mCi).		High-risk: ≥100mCi;
			High risk: 100 mCi				Intermediate-risk: the benefit of RAT≥100mCi is unclear

* routinely recommend.

^a^ Intermediate-risk with any of the following: aggressive histology, minor extrathyroidal extension, vascular invasion, or >5 involved lymph nodes(0.2-3 cm).

^b^ High-risk with any of the following: Gross extrathyroidal extension, incomplete tumor resection, distant metastases or lymph node >3 cm.

^c^ Intermediate-risk with any of the following: advanced age, aggressive histologies, increasing volume of nodal disease, extranodal extension, multiple N1 and/or lymph node metastases outside the central neck.

^d^ Extensive vascular invasion (minimally invasive HCC is characterized as an encapsulated tumor with microscopic capsular invasion and without vascular invasion).

Dx-WBS, ^131^I diagnostic whole-body scan; MVI, minor vascular invasion; cLNM, central lymph node metastases; ETE, extrathyroidal extension; EVI, extensive vascular invasion; CT, computed tomography; MRI, magnetic resonance imaging.

By reviewing the relevant literature above, it is not difficult to perceive that clinicopathological features have been the major consideration in RAT decision-making among guidelines. However, clinicopathological features alone may not be enough to accurately predict the recurrent/metastatic risks of patients, as the real-time status of patients may be altered by initial therapy, urging the consideration of postoperative disease status.

### 1.2 Postoperative disease status-guided RAT

#### 1.2.1 Pros and cons of postoperative disease status guided RAT

Integrated with real-time biochemical and Dx-WBS evaluation,postoperative disease status appears more essential for clinicians to determine if RAT is needed. Serum Tg or Tg-antibody (TgAb) levels, as sensitive organ-specific markers, are part of early postoperative disease status evaluation to identify patients who need more aggressive therapy. Studies have confirmed that patients with higher postoperative TSH-stimulated Tg (ps-Tg) (>1–2 ng/mL) at the time of RAI ablation have an increased risk of recurrence (25, 26), in contrast to ps-Tg <1–2 ng/mL as indicators of remission (27, 28). A few studies have been conducted in a large sample with long-term follow-up regarding the correlation between postoperative Tg levels and the clinical benefit of RAT (**Table 3**). A meta-analysis involving 3947 DTC patients in fifteen studies demonstrated that DTC patients with ps-Tg levels less than 10 ng/ml without the influence of TgAb levels are ideally linked to a better prognosis through RAT, and merely 6% of those patients had persistent disease (26). In a multicentre prospective study, 80% DTC patients with ps-Tg levels ≥10 ng/ml, as well as worse clinicopathological features, kept in a non-structurally incomplete response after 5.55 GBq (150 mCi) of RAT, with a median follow-up of 10.6 months (30). Therefore, higher ps-Tg levels (≥10 or 30 ng/ml) which can reflect unsatisfying real-time postoperative disease status, are likely to guide the consideration of RAT (26, 29). While in patients with a lower Tg cut-off value of 0.27 and 1.4 ng/ml (suppressive vs stimulated), RAT failed to improve patients’ prognosis. Therefore, a definite Tg cut-off remains hard to define, and more evidence is needed to determine the optimal Tg levels of suspicious globulinemia which could benefit from RAT (25).

**Table 3:** Clinical outcomes of postoperative disease status guided RAT.

Study	Study design	Number	Follow-up period	Tg cut-off (ng/ml)	Endpoint	Prognosis improved yes/no	Dosage	Benefited subgroups
Richard C Webb et al. (2012) (26)	Meta-analysis	3947	0.6-16y	(Stimulated) 10	Recurrence	Yes	Na	Ps-Tg ≥10 ng/ml
M Brassard et al. (2011) (25)	Retrospective	715	Median 6.2y	(Suppressed) 0.27 after 3 months of RAI ablation; (Stimulated) 1.4 after 9-12 months of RAI ablation;	Recurrence	No	30-100mCi	Na
Handkiewicz-Junak et al. (2007) (29)	Retrospective	235	6.8y (0.4y-33.5y)	(Stimulated)30	Recurrence	Yes	Patients <12y: 2.0-2.5 mCi/kg of body weight; >12y: 60-100mCi	Ps-Tg ≥30 ng/ml in patients ≤18y

Here, only current studies with long-term follow-up and large sample sizes are listed.

ps-Tg, postoperative stimulated Tg.

Also, Dx-WBS was considered as an indispensable modality in evaluating postoperative disease status, for it can provide supplementary information by detecting functional local regional and distant metastatic lesions (31, 32). Accidental findings on DxWBS could lead to changes in clinical management in about 29.4%-53% of the patients, for whom the intention of RAI upgraded from ablation/adjuvant to treatment of know disease (32, 33).

#### 1.2.2 Recommendations from guidelines concerning postoperative disease status guided RAT

The NCCN Guidelines suggested patients who had elevated postoperative unstimulated Tg >5-10 ng/ml but no positive imaging evidence and patients whose postoperative unstimulated Tg levels were normal but with a high risk of recurrence undergo RAT (22). Besides, the significance of Dx-WBS was also emphasized and was recommended by both the 2015 ATA Guidelines and the NCCN Guidelines (3, 22) (**Table 4**).

**Table 4:** Post-operative status guided RAT through guidelines.

	ATA guidelines		2019 ESMO guidelines	NCCN Guidelines		
Indications	2009 ATA guidelines	2015 ATA guidelines	NS	2018 NCCN guidelines	2020 NCCN guidelines	2021 NCCN guidelines
*Post-operative status guided*	NS	NS	NS	Post-operative unstimulated Tg levels (+)	Post-operative unstimulated Tg >5–10 ng/ml^a^	Post-operative unstimulated Tg >5–10 ng/ml^a^
				Dx-WBS (-)		Dx-WBS (-), CT/MRI+
**Dosage**	100-200 mCi	30-150 mCi	50-100 mCi	50-200 mCi	50-100mCi	
		T3&N1: the effectiveness of RAT >150 mCi is uncertain		RAI for known disease (100-200mCi) is embodied in RAT (50-100mCi).		

^A^Tg values obtained 6–12 weeks after total thyroidectomy; Additional cross-sectional imaging (CT or MRI of the neck with contrast and chest CT with contrast) should be considered to rule out the presence of significant normal thyroid remnant or gross residual disease and to detect clinically significant distant metastases).

NS, not stated.

Therefore, postoperative disease status guided RAT seems mainly based on unexplained postoperatively hyperthyroglobulinemia, with assistance of Dx-WBS to exclude functional lesions. Obviously, it could be a complement to clinicopathological features guided RAT.

### 1.3 Response to therapy evaluation guided RAT

#### 1.3.1 Pros and cons of response to therapy evaluation guided RAT

As far as we are concerned, RAT during follow-up is a kind of “empiric therapy”, which is given to patients with an incomplete biochemical response (BIR, by ATA response to therapy evaluation) to the previous 1^st^ RAI therapy. So far, the precise level of serum Tg which to trigger RAT has been uncertain, and most studies take 10 ng/mL as the cut-off value for its proven high predictive value for recurrence. BIR patients usually have good clinical outcomes, and approximately 56-68% of them will be downstaged to no evidence of disease (NED) during follow-up (34), while 8%–17% of BIR patients still develop structurally identifiable disease over 5–10 years of follow-up (34, 35). In addition, if a patient presents a rapidly rising serum Tg level, it is more likely for them to progress to distant metastases and locoregional recurrence (36). The 2015 ATA Guidelines proposed that the purpose of empirical therapy is to locate possible disease and even to treat it, since a wide range (25%-94%) of these patients may reveal uptake on RxWBS (37–40).

Considering the increased diagnostic efficacy under a therapeutic dose, the poor prognosis of distant metastatic patients may be improved by the early detection of unexpected lesions. Regarding the therapeutic efficacy of RAT, taking Tg as a marker to reflect tumor burden, more than half of such patients present a decreasing pattern after RAI administration. Van Tol et al. reported that half of the patients could even achieve a complete remission (CR) (40, 41). However, it should be noted that a spontaneous decline of Tg is not uncommon. Vaisman et al. demonstrated that 34% of BIR patients could transition to NED status without any additional RAI therapy (34). Moreover, the therapeutic effect of RAT for BIR patients seems to be restricted to the biochemical level, and no persuasive evidence for improved survival has been found (38, 41, 42). In addition, Klain M et al. found that the response to empirical treatment at 12 months seems to be related to the clinical outcome. Distant metastases at RxWBS, CR and objective response rate were predictors of both progression-free survival and overall survival (43) (**Table 5**). Thus, the benefit from response to therapy evaluation guided RAT such as “empiric therapy” remains debatable.

**Table 5:** Clinical outcomes of response to therapy evaluation guided RAT.

Study	Study design	Number	Follow-up period	Tg cut-off (ng/ml)	Endpoint		Prognosis improvedyes/no	Dosage	Benefited subgroups
Pacini F et al. (2001) (41)	Retrospective	70	6.7 ± 3.8 y	7-207	Tg levels		Yes	90-150mCi	Lung metastases on RxWBS, (may in those with lymph node metastases on RxWBS)
Tramontin MY et al. (2021) (42)	Retrospective	120	15.5 y	10		Tg levels, ATA response to therapy status and OS	No	100-600mCi	Na
Van Tol KM et al. (2003) (38)	Retrospective	56	Median 4.2 y (0.5-13.5 y)	(Stimulated) 1.7-10700		Suppressed Tg levels and 5-year survival	Yes	150mCi	RxWBS+ group (more additional ^131^I treatment)
Klain M et al. (2019) (43)	Retrospective	100	96 ± 75 m	5		PFS and OS	Yes	165 ± 46 mCi	CR or PR
Kim WG et al. (2010) (44)	Prospective	39	Median 4.2 m (3-104 m)	10		Recurrence and stimulated Tg levels	No	150mCi	Na

Here, only studies with long-term follow-up or large sample sizes are listed.

CR, complete remission; PR, partial remission; SD, stable disease, PD, progressive disease; PFS, progression-free survival; OS, overall survival; RxWBS, post therapy whole-body scan.

#### 1.3.2 Recommendations from guidelines concerning response to therapy evaluation guided RAT

As for the recommended RAI administered activity, an “empirical RAI therapy” of 100-200 mCi may be considered in patients with elevated ps-Tg ≥10 ng/mL or rapidly rising serum Tg levels with negative imaging in the 2015 ATA guidelines. Similarly, the 2018 Chinese Society of Clinical Oncology Guidelines (the 2018CSCO Guidelines) suggested that patients with persistent/recurrent/metastatic disease whose elevated levels were >10 ng/ml without WBS evidence may benefit from ^131^I empirical therapy with weak recommendations (45). The 2021 NCCN guidelines suggested consideration of RAT ≥100 mCi in patients with progressively rising Tg (basal or stimulated) and negative scans, including positron emission tomography (PET) (22). Notably, it emphasized the application of PET scans because they have shown great value in detecting incidental lesions and therefore improving restaging (46). However, there was no treatment instruction for BIR patients in the 2019 ESMO guidelines; only a RxWBS after “therapeutic” activity was suggested if the PET scan was normal (23) (**Table 6**).

**Table 6:** Response to therapy evaluation guided RAT through guidelines.

	ATA guidelines		NCCN guidelines			2018CSCO guidelines
Indications	2009 ATA guidelines	2015 ATA guidelines	2018 NCCN guidelines	2020 NCCN guidelines	2021 NCCN guidelines	
*Response-to-therapy evaluation guided*	NS	Tg levels↑	NS	NS	NS	Tg(+)WBS(-) or Tg(+)^18^F FDG PET/CT(-) (ps-Tg≥10 ng/ml)
**Dosage**	100-200 mCi	30-150 mCi	50-100 mCi	50-200 mCi	50-100mCi	100-200mCi
		T3&N1: the effectiveness of RAT >150 mCi is uncertain		RAI for known disease (100-200mCi) is embodied in RAT (50-100mCi).		>150mCi should be avoided in patients≥70y

WBS, whole-body scan, especially ^131^I-diagnostic whole-body scan, also as Dx-WBS; ps-Tg, post-operative stimulated Tg; FDG PET/CT, fluorodeoxyglucose positron emission tomography; NS, not stated.

Therefore, response to therapy evaluation guided RAT is mainly based on the undesirable BIR to prior RAI therapy, meanwhile, it should be performed with caution only when the expected efficacy will outweigh its side effects. We can expect that, it could be further clarified along with the evolving imaging techniques.

## 2 Future directions of RAT

Along with the rapidly evolving techniques, the theranostic value of nuclear medicine molecular imaging has been highlighted as an indispensable modality in accurate pre-RAI assessment and therapeutic response of RAI prediction, thus assisting in refining the aim of RAT (47).

### 2.1 Promising value of theranostic imaging

**Figure 1:**
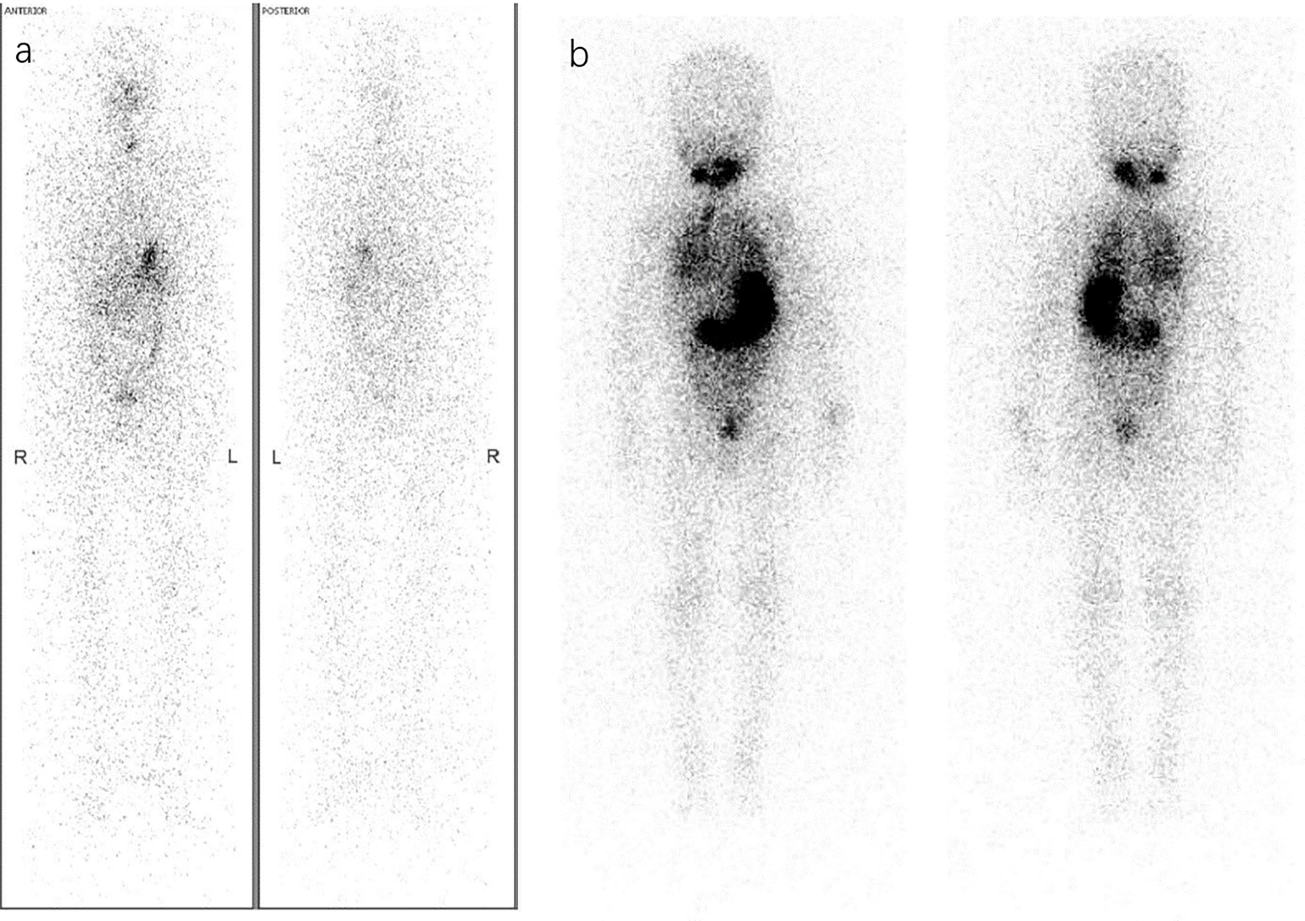
Different RAT considerations *via* Dx-WBS. **(A)** A 36-year-old woman was diagnosed with PTC (pT1bN1bM0, ATA intermediate-risk) by pathology after thyroidectomy and lymph node dissection. Considering both the postoperative disease status of ps-Tg<0.04 ng/ml and A-Tg 111 IU/ml with a decreasing trend and Dx-WBS findings that did not indicate suspicious concealed lesions, she may not benefit from RAT. Therefore, she received TSH suppression therapy instead of RAT. **(B)** A 4-year-old boy under thyroidectomy was confirmed pathologically to have PTC (pT1aN1aM0, ATA low-risk) in 2008. Surprisingly, Dx-WBS revealed wide uptake of ^131^I in the lungs, whereas preoperative CT did not. Through 3 times of 60 mCi RAI, he obtained and has maintained an excellent response (ER) in recent years (1^st^ RAT: TSH >150 μIU/mL, Tg 91 ng/ml, A-Tg 13.25 IU/ml; 2^nd^ RAT: TSH >150 μIU/mL, Tg 9.4 ng/ml, A-Tg 10 IU/ml; 3^rd^ RAT: TSH >150 μIU/mL, Tg 3.4 ng/ml, A-Tg 10 IU/ml; the latest follow-up: TSH 0.244 μIU/mL, Tg<0.04 ng/ml, A-Tg 15 IU/ml).

Radioiodine-WBS (^131^I,^123^I,^124^I), which is the best example of the so-called “theranostics”, can reflect sodium/iodide symporter (NIS) expression and iodine metabolism from both diagnostic and therapeutic perspectives and will play a complementary role in the abovementioned 3-factor guided RAT. Of note, despite the informative value of demonstrating the risk of persistence/recurrence/metastasis, a single clinicopathologic feature itself may not be enough to dominantly direct the strategy of RAT. For instance, by integrating ^131^I theranostics as a dynamic assessment modality, those with high pathological risk features but downstaged by adequate prior therapy may be spared from unnecessary aggressive RAT, while those with a low risk stratified by clinicopathologic features may be upstaged and be more likely to benefit from RAT (**Figure 1**). Likewise, although postoperative serum Tg levels are predictive for evaluating postoperative disease status, they might be confused by either residual thyroid cancer or remnant tissue and TSH/TgAb levels during measurement (48, 49).

**Figure 2:**
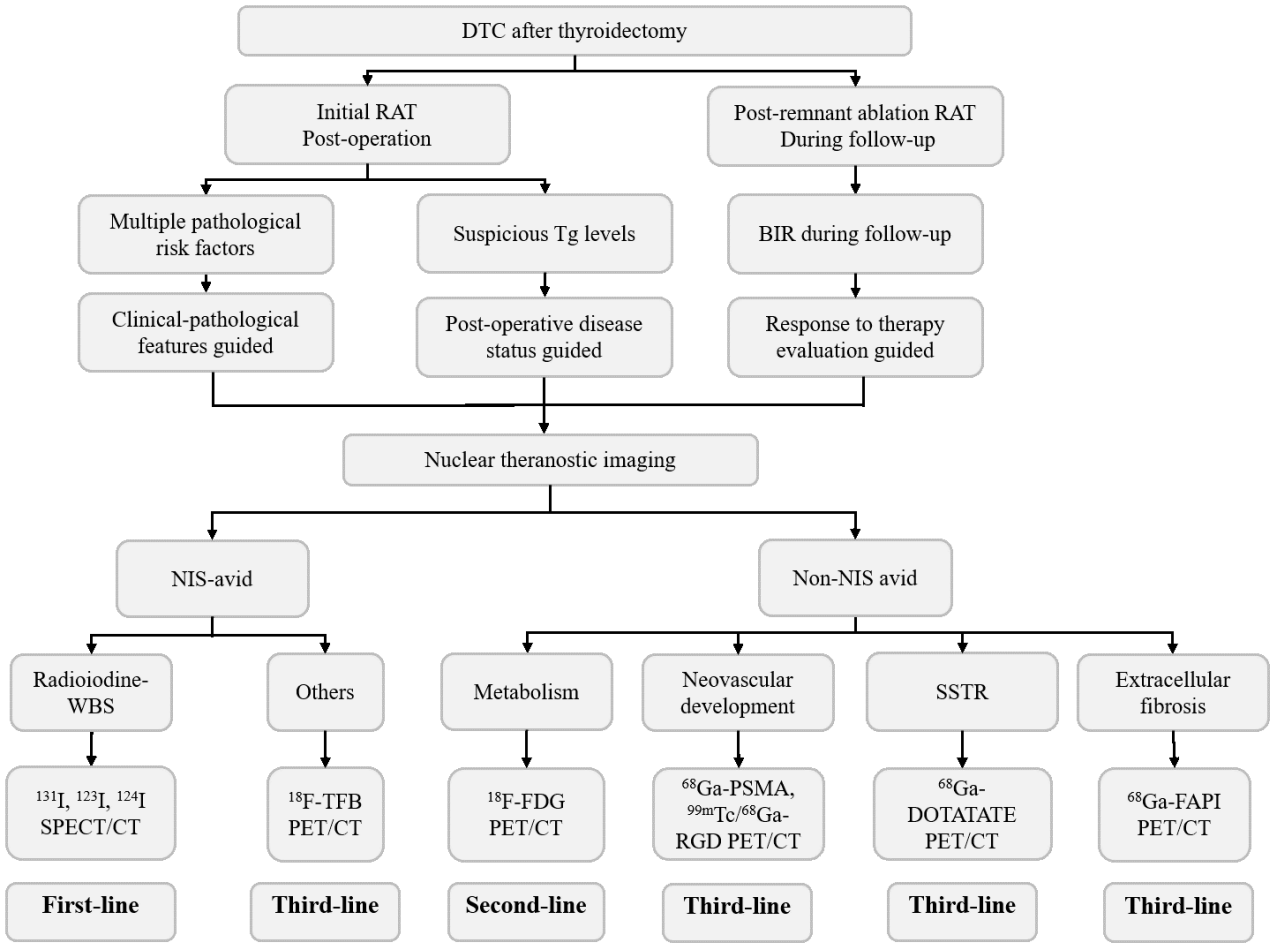
Diagram of the RAT decision-making strategy in DTC.

As a result, the traditional factor-guided RAT may be further refined by incorporating theranostic imaging evidence, including Dx-WBS, for identifying NIS-avid lesions and other modalities for confirming the actual evidence of tumor existence (**Figure 2**). Recent studies suggested that apart from iodine, other NIS-targeted imaging modalities, such as ^18^F-tetrafluoroborate (^18^F-TFB), may provide incremental value for sensitive detection of RAI-avid lesions (50). Under such circumstances, the conception of RAT may be altered from suspected lesions to known lesions. When it comes to non-NIS-avid lesions, they may be detected by ^18^F-FDG PET/CT at the level of tumor metabolism and reflected by ^68^Ga-prostate-specific membrane antigen (^68^Ga-PSMA) PET/CT as well as ^99m^Tc/^68^ Ga-arginine-glycine-aspartic acid (^68^Ga-RGD) PET/CT at the level of neovascular development ^68^.Ga-DOTATATE and ^68^Ga-fibroblast-activation-protein inhibitors (^68^Ga-FAPI) PET/CT can demonstrate the expression of somatostatin receptors (SSTRs) and extracellular fibrosis of tumors, respectively (51–55). In clinical practice, lesions with adequate NIS expression (NIS-avid) will inspire clinicians to prescribe a therapeutic rather than a merely adjuvant dosage of ^131^I, while others (non-NIS-avid) may be transferred to other treatments based on their individual molecular characteristics.

Notably, the *theranostics* mentioned above are merely focused on the diagnostic superiority of molecular nuclear theranostic imaging. Those who are diagnosed as BIR by traditional anatomical modalities may be additionally identified as having a structural incomplete response (SIR) by means of nuclear molecular theranostics. The more thorough the imaging studies are, the fewer BIR patients are left. Thus, comprehensive structural and functional imaging studies could be expected in the future.

### 2.2 Genetic background of theranostics

The genetic background plays a vital role in the underlying tumor behavior and theranostic imaging results. Studies have confirmed that the expression of NIS can predict responsiveness to RAI therapy to some degree (56, 57), and Xing et al. proved that once DTC carries the *BRAF^V600E^* mutation, it may manifest a relatively low level of NIS expression, thus leading to unsatisfactory radioiodine uptake (non-RAI avidity) and a high risk of recurrence (56). The authors also previously reported that if *BRAF^V600E^* mutation was concomitant with other oncogenic mutations such as *TERT* mutations, it would have a robust synergistic impact on the aggressiveness of DTC, and the benefit from RAI therapy was very limited (57, 58). Moreover, coexisting *BRAF^V600E^* mutation and *PIK3CA, TP53*, and *AKT*1 mutations were also identified as predictors of a less favourable clinical outcome by recent studies (59–62). Thus, the heterogeneous mutations of genes can explain the differences in tumor morphology, gene expression and clinical features of every individual, which are expected to become prognostic molecular markers for RAT decision-making. Therefore, the impact of mutations on disease evolution remains to be further explored.

Nevertheless, no accurate therapeutic response evaluation system for RAT has been established owing to a lack of strong evidence, and few data can help in forming a consensus on the detailed indications and clinical outcomes of RAT. Well-designed, prospective randomized controlled trials are urgently needed to offer an accurate therapeutic response evaluation system and avoid confusing decision-making based on observational and low-quality studies with diverse outcomes.

## 3 Conclusions

**Figure 3:**
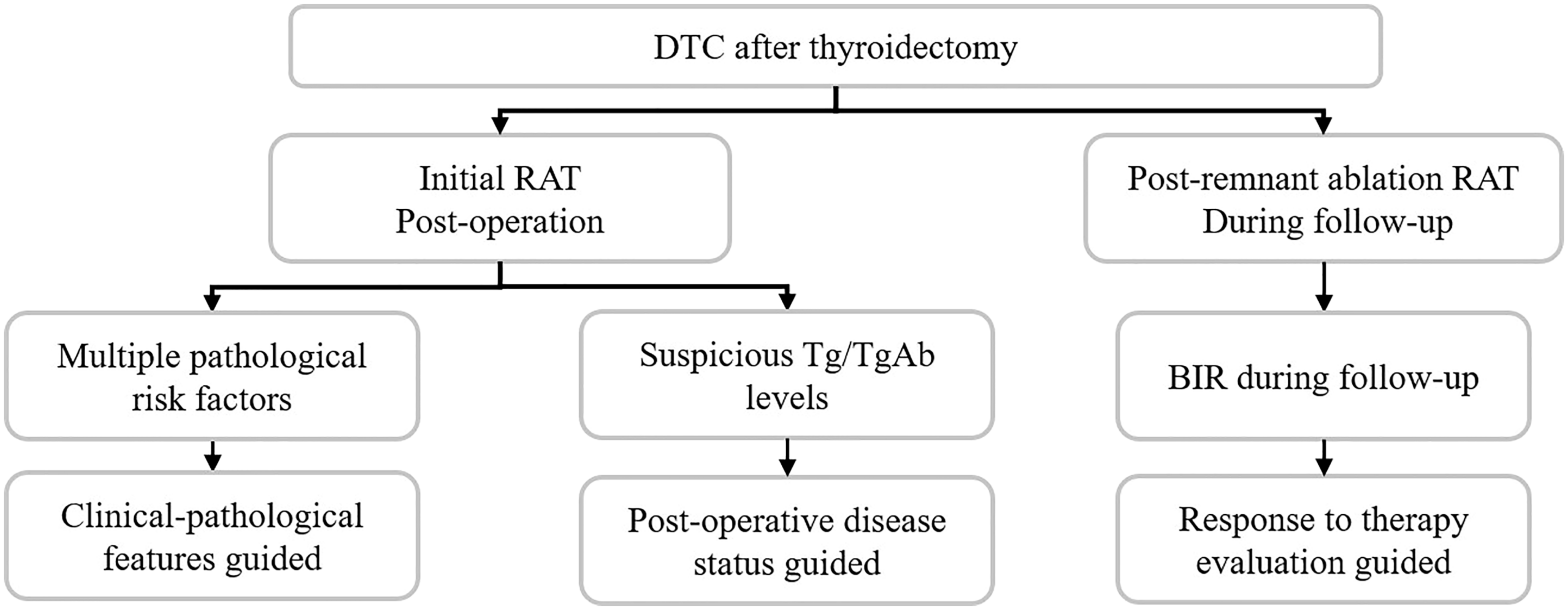
Current factors guided RAT decision-making.

In this review, we have summarized three aspects of RAT indications (**Figure 3**), which can help advance our understanding of RAT in a complementary way. Although consensus is not yet reached on certain RAT guiding features, it offers a new perspective in clinical RAT decision-making. Nuclear medicine imaging and genetic background may help refine the RAT conception and clinical practice in the future. Furthermore, well-designed studies with strong evidence are urgently needed.

## Author contributions

Y-QS, DS, and Y-SL contributed to conception and design of the study. Y-QS gathered evidence and wrote the first draft of manuscript. DS wrote sections of the manuscript. All authors contributed to the article and approved the submitted version.

## Funding

This work was supported by the CAMS Innovation Fund for Medical Sciences (CIFMS) (No. 2020-I2M-2-003); Project on Inter-Governmental International Scientific and Technological Innovation Cooperation in National Key Projects of Research and Development Plan (Grant No. 2019YFE0106400); National High Level Hospital Clinical Research Funding; National Natural Science Foundation of China (No. 81771875 ); the CSCO-Hengrui Research Foundation (No. Y-HR2018-143, Y-HR2018-144.

## Conflict of interest

The authors declare that the research was conducted in the absence of any commercial or financial relationships that could be construed as a potential conflict of interest.

## Publisher’s note

All claims expressed in this article are solely those of the authors and do not necessarily represent those of their affiliated organizations, or those of the publisher, the editors and the reviewers. Any product that may be evaluated in this article, or claim that may be made by its manufacturer, is not guaranteed or endorsed by the publisher.
