# Supplementary material for: Radioiodine adjuvant therapy in differentiated thyroid cancer: An update and reconsideration
Source: Front Endocrinol (Lausanne). 2022 Nov 30;13:994288. doi: 10.3389/fendo.2022.994288 (PMC9747769; doi:10.3389/fendo.2022.994288)
Supplement: Supplementary file 1 [file Table_1.docx]

Supplementary Table 1. Indications of guidelines in clinicopathological features.

|  | **ATA Guidelines** | | **2019 ESMO Guidelines** | **NCCN Guidelines** | | | **2018CSCO Guidelines** |  |
| --- | --- | --- | --- | --- | --- | --- | --- | --- |
|  |  |  |  |  |  |  |  |  |
| **Indications** | **2009 ATA Guidelines** | **2015 ATA Guidelines** |  | **2018 NCCN Guidelines** | **2020 NCCN Guidelines** | **2021 NCCN Guidelines** |  |  |
| *Clinicopathological features guided* | Tumor >1.5 cm  With post-operatively residual disease | (Updated on 2009 ATA Guidelines)  1.Intermediate- ^a^ and high-risk^b^*****  2.BRAF^V600E^ | Intermediate-and high-risk  (Updated on 2015 ATA Guidelines)  1.Intermediate-risk: Tumor-related symptoms  2.High-risk: TERT(+) and BRAF^V600E^ (+) | Clinicopathologic findings(+), Dx-WBS(-):  1.Tumor >2cm;  2.MVI (+)  3.cLNM(+)  4.Microscopic margins(+)  5.ETE(+) | (Updated on 2018NCCN Guidelines)  1.RAT selectively recommended:  Tumor 2–4 cm  2.RAT recommended*:  Tumor >4 cm  ETE(+)  EVI^c^  Bulky or LNM >5 | Dx-WBS(-), CT/MRI+ | NS |  |
|  |  |  |  |  |  |  |  |  |
|  |  |  |  |  |  |  |  |  |
|  |  |  |  |  |  |  |  |  |
|  |  |  |  |  |  |  |  |  |
|  |  |  |  |  |  |  |  |  |
|  |  |  |  |  |  |  |  |  |
|  |  |  |  |  |  |  |  |  |
| **Dosage** | 100-200 mCi | 30-150 mCi | Low risk except for pT1a:≥30 mCi | 50-100 mCi | 50-200 mCi | 50-100mCi | 100-200mCi |  |
|  |  |  |  |  |  |  |  |  |
|  |  |  |  |  |  |  |  |  |
|  |  | T3&N1: the effectiveness of RAT >150 mCi is uncertain | Intermediate risk: 30-100 mCi |  | Known disease RAI (100-200mCi) is embodied in RAT (50-100mCi). |  | >150mCi should be avoided in patients≥70y |  |
|  |  |  | High risk: 100 mCi |  |  |  |  |  |

*: routinely recommend

a: 1.1 Intermediate-risk with any of the following: aggressive histology, minor extrathyroidal extension, vascular invasion, or >5 involved lymph nodes(0.2-3 cm)

b: 1.2 High-risk with any of the following: Gross extrathyroidal extension, incomplete tumor resection, distant metastases or lymph node >3 cm

c: Extensive vascular invasion (minimally invasive HCC is characterized as an encapsulated tumor with microscopic capsular invasion and without vascular invasion)

Abbreviations: Dx-WBS, ^131^I diagnostic whole-body-scan; MVI, minor vascular invasion; cLNM, central lymph node metastases; ETE, extrathyroidal extension; EVI, extensive vascular invasion; CT, computed tomography; MRI, magnetic resonance imaging; NS, not stated.

Supplementary Table 2. Post-operative status guided RAT through guidelines.

|  | **ATA Guidelines** | | **2019 ESMO Guidelines** | **NCCN Guidelines** | | |  |
| --- | --- | --- | --- | --- | --- | --- | --- |
|  |  |  |  |  |  |  |  |
| **Indications** | **2009 ATA Guidelines** | **2015 ATA Guidelines** | NS | **2018 NCCN Guidelines** | **2020 NCCN Guidelines** | **2021 NCCN Guidelines** |  |
| *Post-operative status guided* | NS | NS |  | Post-operative unstimulated Tg levels(+) | Post-operative unstimulated Tg >5–10 ng/ml^a^ | NS |  |

a: Tg values obtained 6–12 weeks after total thyroidectomy; Additional cross‑sectional imaging (CT or MRI of the neck with contrast and chest CT with contrast) should be considered to rule out the presence of significant normal thyroid remnant or gross residual disease and to detect clinically significant distant metastases.)

Abbreviations: NS, not stated.

Supplementary Table 3. Response to therapy evaluation guided RAT through guidelines.

|  | **ATA Guidelines** | | **NCCN Guidelines** | | | **2018CSCO Guidelines** |  |  |
| --- | --- | --- | --- | --- | --- | --- | --- | --- |
|  |  |  |  |  |  |  |  |  |
| **Indications** | **2009 ATA Guidelines** | **2015 ATA Guidelines** | **2018 NCCN Guidelines** | **2020 NCCN Guidelines** | **2021 NCCN Guidelines** |  |  |  |
| *Response-to-therapy evaluation guided* | NS | Tg levels↑ | NS | NS | NS | Tg(+)WBS(-) or Tg(+)^18^F FDG PET/CT(-)  (ps-Tg≥10 ng/ml) |  |  |
| **Dosage** | 100-200 mCi | 30-150 mCi | 50-100 mCi | 50-200 mCi | 50-100mCi | 100-200mCi |  |  |
|  |  |  |  |  |  |  |  |  |
|  |  |  |  |  |  |  |  |  |
|  |  | T3&N1: the effectiveness of RAT >150 mCi is uncertain |  | Known disease RAI (100-200mCi) is embodied in RAT (50-100mCi). |  | >150mCi should be avoided in patients≥70y |  |  |
|  |  |  |  |  |  |  |  |  |
| Abbreviations: WBS, whole-body-scan, especially ^131^I-diagnostic whole body scan, also as Dx-WBS;  ps-Tg, post-operative stimulated Tg; FDG PET/CT, fluorodeoxyglucose positron emission tomography; NS, not stated. | | | | | | | |  |
